# Non-volatile Clocked Spin Wave Interconnect for Beyond-CMOS Nanomagnet Pipelines

**DOI:** 10.1038/srep09861

**Published:** 2015-05-08

**Authors:** Sourav Dutta, Sou-Chi Chang, Nickvash Kani, Dmitri E. Nikonov, Sasikanth Manipatruni, Ian A. Young, Azad Naeemi

**Affiliations:** 1 School of Electrical and Computer Engineering, Georgia Institute of Technology, Atlanta, GA 30332 USA; 2 Components Research, Intel Corporation, Hillsboro, OR 97124 USA

## Abstract

The possibility of using spin waves for information transmission and processing has been an area of active research due to the unique ability to manipulate the amplitude and phase of the spin waves for building complex logic circuits with less physical resources and low power consumption. Previous proposals on spin wave logic circuits have suggested the idea of utilizing the magneto-electric effect for spin wave amplification and amplitude- or phase-dependent switching of magneto-electric cells. Here, we propose a comprehensive scheme for building a clocked non-volatile spin wave device by introducing a charge-to-spin converter that translates information from electrical domain to spin domain, magneto-electric spin wave repeaters that operate in three different regimes - spin wave transmitter, non-volatile memory and spin wave detector, and a novel clocking scheme that ensures sequential transmission of information and non-reciprocity. The proposed device satisfies the five essential requirements for logic application: nonlinearity, amplification, concatenability, feedback prevention, and complete set of Boolean operations.

Despite the unprecedented success of the complementary metal-oxide semiconductor (CMOS) in terms of dimensional scaling, recent International Technology Roadmap for Semiconductors (ITRS) projections suggest the apparent conclusion of the scaling trend due to fundamental physical limitations 1,2,3,4. This leads to an aggressive global search for novel devices and alternative state variables. In particular, the possibility of using electron spin as a computational state variable for logic devices and circuits has been the focus of active research 2,5,6. Among these spin-based devices, computation based on spin wave, a collective oscillation of electron spins doing precession motion around a fixed direction of magnetization, has drawn a lot of attention owing to the following advantages: (i) transmission of information without any charge transfer, (ii) superposition of spin waves providing ability to perform logical operations, (iii) no stand-by power requirement, and (iv) interaction between the spin wave bus and other devices being accomplished via magnetic coupling 7,8,9,10,11,12,13,14,15. Recently, all-magnon based circuits namely majority gate 16,spin-wave multiplexer 17 and magnon transistor in which the source-to-drain magnon current is controlled by the injection of magnons into the transistors gate 18 have been proposed for all magnon data processing. Designing of a complete spin wave device (SWD) would require the following characteristics: efficient excitation and detection of spin waves, amplification of spin waves to compensate the attenuation of the propagating signal through long chain of concatenated gates, non-volatile storage of information, ability to drive the next stage (concatenability) and non-reciprocity.

In this paper, we propose a comprehensive scheme for information transmission via spin waves. The proposed structure has some similarities with the previous work in terms of integrating magneto-electric (ME) cells with spin wave bus (SWB) 19,20,21,22. However, unlike in 21 where the logic is stored as a canted magnetization state of the ME cell and a propagating spin wave can switch the canted magnetization from one state to the other, here we propose a 180° switching of the magnetization by the propagating spin wave by introducing a meta-stable magnetization state via the ME effect. It is well-known that spin waves can cause small fluctuations of the magnetization around the equilibrium condition. Hence, spin waves on their own can not provide enough energy to the magnet to cross the barrier and switch from one in-plane stable state to the other. However, one can imagine a case where the ME effect can modify the energy profile of the magnet, making it go to a meta-stable out-of-plane state (90° switching) and once the ME effect is removed, the phase of the propagating spin waves can deterministically make the magnet go to either one of the stable in-plane configurations (another 90° switch). Thus this scheme provides a more stable non-volatile memory element even when the effect of thermal noise is considered. While similar approach to use a meta-stable magnetization state for spin wave detection has been suggested in a simplistic way in 23, ours is the first to take into consideration the essential characteristics like non-reciprocity and concatenability missing in the previous works and requires a carefully designed clocking scheme.

A major drawback of the spin waves is the exponential decay of the signal amplitude 19. Hence, the realization of a feasible long multi-staged spin wave based circuitry would require either spin wave amplifiers to boost the signal amplitude 20 or convert spin wave signal to voltage signal at the end of each stage. The repeated spin-to-charge conversion would require extra circuitry that defeats the advantage of having spin based devices. In the proposed scheme, we use the 90° switching of magnetization from a stable in-plane to a meta-stable out-of-plane state via ME effect to create new spin waves for transmitting signal to the next stage. This mechanism ensures an automatic amplification of spin wave signal at the end of each stage. Concatenability is ensured by designing the input and output of each stage in a similar fashion such that the output of one stage can serve as the input of the next stage. The bistability of the ME cell magnet storing the information provides the non-linearity for logic application. Another key requirement for logic implementation is non-reciprocity, ie., controlling the direction of signal flow such that the output gets affected by the input and not the reverse. Recently, the non-reciprocal behavior of magnetostatic surface spin waves has been investigated for logic applications 24,25. The origin of the non-reciprocity was the interference of the spin waves produced by two different components (in-plane and out-of-plane) of the magnetic field due to a current flowing through the waveguide. Here, we propose a novel clocking scheme which takes care of non-reciprocity while ensuring sequential detection, storage and transmission of information from one stage to the next stage in a cascaded SWD. As will be discussed later, the appropriate choice of the clock period results in the device acting as a buffer (PASS gate) or an inverter (NOT gate). This serves as a building block for designing majority gates and other logic gates, thus providing a complete set of Boolean operations. Our proposed device thus ensures the five essential requirements for logic application: nonlinearity, amplification, concatenability, feedback prevention, and complete set of Boolean operations 2.

Figure 1 (a) shows the schematic of a clocked three-stage cascaded SWD that can act as a chain of inverter or buffer. The structure consists of three main parts: (a) a charge to spin (C-S) converter which transforms electrical signal to spin wave signal at the beginning of the first stage, (b) intermediate ME cells that act as repeaters for spin wave detection, transmission and non-volatile storage, and (c) spin wave interconnects or spin wave buses (SWBs) that act as conduits for information transmission. The sequential switching of the C-S converter and the spin wave repeaters is accomplished by applying voltage pulses between the ground layer and the respective bottom metallic layers as shown by the clocks 1–4. The input data is applied in the form of current pulses (positive current represents bit “1” while negative current represents bit “0”) to the top metal electrode of the C-S converter as shown by the STT clock.

The working principle of the SWD is similar to that of charge-coupled device (CCD) used extensively in digital media. The functionalities of the basic building block in SWD, ie. the ME cell, is analogous to that in CCD, ie., receive a token of information from a previous cell, hold the token for a time without appreciable loss, and pass the token to the next cell. The clocking scheme of the ME cells designed for sequential transmission of information is comparable to the n-phase clocking technique used in CCD to encourage the charge packets to move cell to cell in a bucket-brigade style.

## Results

### Charge-to-Spin Converter

We start by analyzing the C-S converter which is meant to perform two major operations: (a) translate information (bit “1” or “0”) from electrical domain to spin domain by switching to one of the two low-energy stable magnetization states. This switching must be determined by a voltage or current dependent external force like a spin-torque where a positive or negative spin current switches magnetization to two opposite states. Other possible options can be via a voltage-controlled easy-axis rotation where the polarity of the voltage governs a clockwise or anti-clockwise rotation or via exchange-bias where depending on the polarity of the applied voltage, a positive or negative exchange bias may favor one of the two possible final states, and (b) transmit the stored information by exciting spin waves with the correct phases (0 or π), where a 0-phase corresponds to bit “1” and π-phase corresponds to bit “0”.

**Figure 1:**
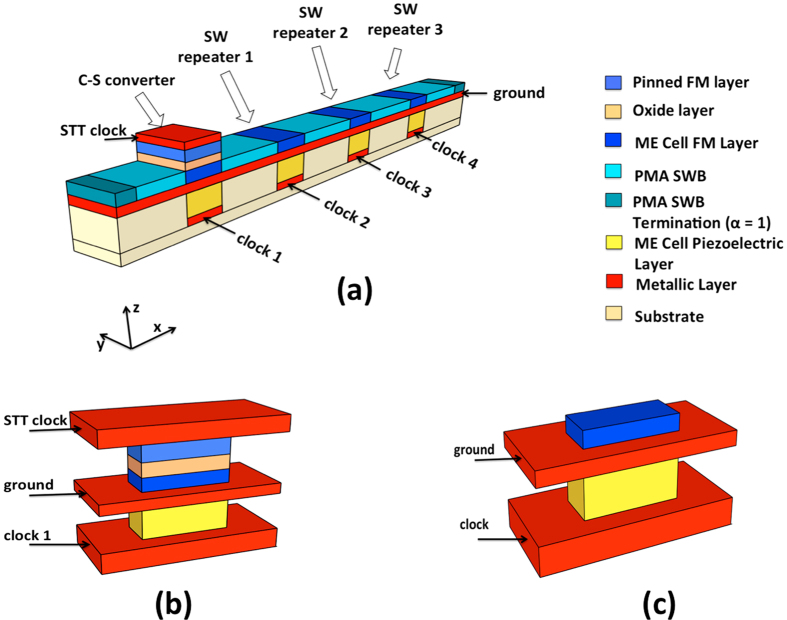
Illustration of a clocked three-stage cascaded spin wave device comprising of: a charge to spin (C-S) converter, intermediate spin wave repeaters and spin wave interconnects. (**a**) Detail schematics of the C-S converter and spin wave repeater are shown in (**b**) and (**c**). The sequential switching of the converter and the repeaters is accomplished via application of clocks 1-4 while the input data is applied in the form of current pulses using the STT clock.

**Figure 2:**
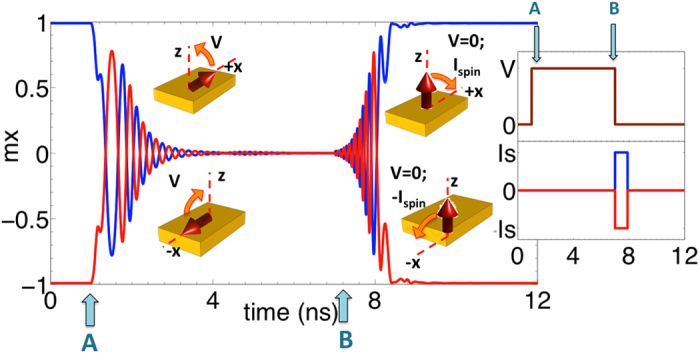
Magnetization dynamics of the C-S converter. The inset shows the sequence of applied voltage across the piezoelectric layer and spin current through the MTJ. The rising edge of the clock (point A) denotes the instance of information transmission by exciting spin waves via application of voltage, while the falling edge of the clock (point B) represents storage of information via application of spin-torque. The stored bit will be transmitted in the next clock cycle. The polarity of the injected spin current determines the final magnetization state as shown by the blue and red curves.

We consider a heterostructure comprising of a spin-transfer-torque random access memory (STT-RAM) built on top of a piezoelectric material, similar to the scheme proposed by A. Khan et. al. 26, for our C-S converter design as shown in Fig. 1 (b). The magnetization of the fixed layer is kept pinned in the+x−direction while the free layer has two stable magnetization states along the + or −x directions, determined by the shape anisotropy. A voltage applied across the middle and the bottom metallic layer creates an isotropic or biaxial in-plane strain in the piezoelectric layer. This in-plane strain gets transferred through the thin middle metallic layer to the ferromagnetic free layer above it creating an effective strain-induced out-of-plane perpendicular magnetic anisotropy. If this anisotropy field becomes higher than the out-of-plane demagnetizing field, the magnetic easy-axis rotates out-of-plane causing an out-of-plane switching of magentization. This switching dynamics creates propagating spin waves with the information being encoded into the phase of the waves. We assume that magnetization rotation from +x to z direction creates spin waves with phase 0 while that from -x to z creates waves with phase π. When the voltage is switched off, the strain is removed causing the magnetization to go in-plane. It is interesting to note that upon the removal of the strain, the magnetization has equal possibility of falling back to +x or −x dirction. However, if a small spin current is passed though the free layer for a short duration, it can deterministically put the magnet in one of the two stable states depending on the polarity of the spin current. This can be achieved through the top magneto-tunnel junction (MTJ) structure. The polarity of the injected spin current determines the final magnetization state of the free layer. Figure 2  shows the magnetization dynamics of the C-S converter upon application of voltage and spin current. It is to be noted that the magnetization dynamics during the storage of information via STT also creates propagating spin waves; however, our proposed clocking scheme ensures that they do not affect the the next stage repeater which is explained later.

### Spin Wave Repeater

**Figure 3:**
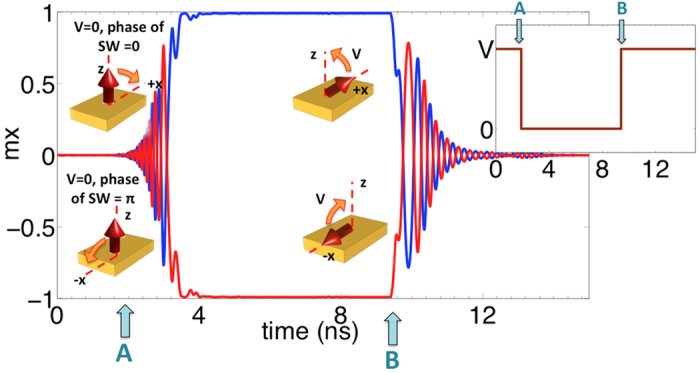
Magnetization dynamics of the spin wave repeater upon application of the voltage across the piezoelectric layer of the ME cell. The falling edge of the clock (point A) denotes the detection of the spin wave signal by turning off the voltage. The phase of the spin wave dictates the direction of tilting of the magnetization. The rising edge of the clock (point B) denotes the transmission of the stored information by exciting spin waves via the application of the voltage.

Next, we analyze the design of the spin wave repeater, aimed to achieve the following three functionalities: (a) receive/detect the spin wave signals propagating from the previous stage, (b) store the information encoded in the phase of the detected spin waves in a magnetization state (non-volatile memory), and (c) transmit the information to the next stage by exciting new spin waves with the correct phase. We design the repeater as a ME cell consisting of a bottom electrode, a piezoelectric layer, a top electrode, and a ferromagnetic (FM) layer which also serves as a part of the spin wave interconnect as shown in Fig. 1 (c). The working of the repeater is similar to that of the C-S converter except that instead of the spin-polarized current determining the storage of an information bit, the phase of the propagating spin waves is used. The FM layer has two low-energy stable in-plane magnetization states along the +or −x direction, favored by the shape anisotropy of the structure. An applied voltage across the top and bottom electrodes causes a biaxial in-plane strain in the piezoelectric layer. This in-plane strain gets coupled to the FM layer creating a strain-induced out-of-plane magnetic anisotropy. Above a critical strain (or induced-anisotropy), the magnetic easy-axis rotates out-of-plane causing an out-of-plane switching of magnetization. Once out-of-plane, the magnetization is continued to be held in the meta-stable state via application of voltage until the spin wave signal propagating from the previous stage reach the repeater. Upon arrival, the voltage is turned off causing the magnetization to relax back to the in-plane configuration, the final state being determined by the phase of the propagating waves. A zero phase causes the magnetization to fall from z to+x direction while a π phase gives -x as the final state. This mechanism describes the detection of the spin wave signal. Once the magnetization falls in-plane and the voltage is removed, it stays in the final state thereby acting as a non-volatile memory element storing either a bit “1” (+x magnetization direction) or a bit “0” (−x magnetization direction). In order to transmit the stored information (bit “1” or “0”) to the next cascaded stage, the voltage is again turned on making the magnetization go from the in-plane (+x or −x direction) to the out-of-plane meta-stable state, thus exciting spin waves with the correct phase. Figure 3 shows the dynamics of the spin wave repeater. It must be noted that the magnetization dynamics during the detection of spin waves also creates propagating spin waves; however, as stated earlier our proposed clocking scheme ensures they do not affect the dynamics of the next stage repeaters.

### Clocking Scheme

**Figure 4:**
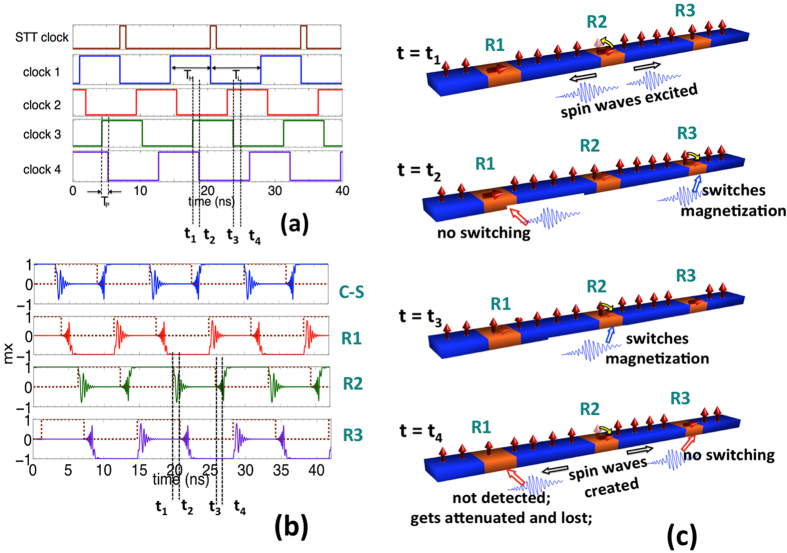
The proposed clocking scheme which enables a sequential transmission of information and non-reciprocity. (**a**) The rising edge of the clock represents the excitation of spin waves while the falling edge represents the detection and storage of signal. *T*_*P*_ denotes the propagation delay of the spin waves from one ME cell to the next while *T*_*H*_ and *T*_*L*_ represents the time a clock stays high or low. (**b**) Shows the corrsponding magnetization dynamics of the C-S converter and repeaters R1, R2 and R3. (**c**) Illustration of the working of the clocking scheme showing a pictorial representation of the spatial variation of the magnetization at different snapshots of time.

The non-reciprocity and the sequential transmission of information from one stage to the next in a SWD is taken care of by introducing a novel clocking scheme shown in Fig. 4(a). External CMOS circuitry can be designed to generate the clocking signals that toggle between the ground and supply voltage, and have a clock skew between each other; however, the design and analysis of such circuitry is beyond the scope of this paper. Both the C-S converter and the spin wave repeaters are designed as edge-triggered elements that transmit and receive signals at the rising and falling edges of the clock. The rising edge of the clock represents the excitation of spin waves for information transmission while the falling edge represents the detection and storage of signal. Note that both the rising and falling edges of the clock (magnetization going in-plane to out-of-plane and out-of-plane to in-plane) excite spin waves that can travel in both the forward and backward directions.

The basic operating principle of the clocking scheme is shown in Fig. 4(c). At time *t = t*_*1*_ when the ME cell R2 is exciting spin waves by switching from in-plane to out-of-plane, the previous stage ME cell R1 maintains an in-plane stable configuration. Since the spin waves propagating in the PMA SWB are in reality only the precessional rotation of the out-of-plane magnetization, having an in-plane magnetization of R1 blocks the waves from further propagation as well as does not affect the state of the R1. On the other hand, the next stage ME cell R3 is maintained in an out-of-plane meta-stable condition by applying a voltage, waiting for the spin waves to arrive and being detected. As soon as the waves reach R3 at time *t = t*_*2*_, the voltage on R3 is removed allowing the magnetization to go in-plane, the switching direction (*+/−*x) dictated by the phase of the incoming waves.

At time *t = t*_*3*_ when R2 is detecting the spin waves, R3 maintains an in-plane stable state thus blocking the forward propagating spin waves created during detection by R2. Since R1 has to be maintained in an out-of-plane meta-stable state in order to detect the spin waves that would be created by C-S (stage previous to R1), the backward propagating spin waves from R2 pass throughout R1 without getting detected. Eventully the waves get attenuated and are lost.

The detection and storage of information in the C-S converter or the spin wave repeater is achieved by maintaining the magnetization of the ME cell in the out-of-plane meta-stable state via application of voltage until an external force in the form of STT or propagating spin wave signal is available to drive the magnetization to a stable in-plane (+/−x) state. In other words, the spin current pulse should go high or the spin wave signal propagating from the previous cell should reach the current cell at the moment the electric field is switched off. We carefully design the clocking scheme and choose the clock period such that this criterion is always fulfilled. Hence, the indeterminate state of magnetization in the absence of spin wave signal or STT after the electric field is switched off is avoided.

The delay between the rising edge of one clock and the falling edge of the next clock (*t*_1_-*t*_2_) is equal to the propagation delay *T*_*P*_ of the spin waves from one ME cell to the next and has to be carefully sized for designing the clocking scheme. Section S2 of the supplementary information further explains the way of choosing the value of *T*_*P*_. The presence of the delay *T*_*P*_ and the requirements for non-reciprocity leads to a negative clock skew and the need for having multiple clocks. Further details about choosing the high time *T*_*H*_ and the low time *T*_*L*_ for the clocking scheme has been explained in the supplementary section S3.

### Single stage device operation

The scheme of maintaining the magnetization in a meta-stable state and detecting the spin waves upon it’s arrival is sensitive to several factors, some key parameters being the propagation time *T*_*P*_ of the spin waves, which in turn depends on the length of the SWB interconnect, and the material parameters like the gilbert damping coefficients α, saturation magnetization *M*_*S*_, exchange stiffness *A*_*exch*_ and material anisotropies *K* of the SWB interconnect and ME cell. The clock period and clock skew are chosen with respect to *T*_*P*_ as mentioned in section S3 of supplementary information.

**Figure 5:**
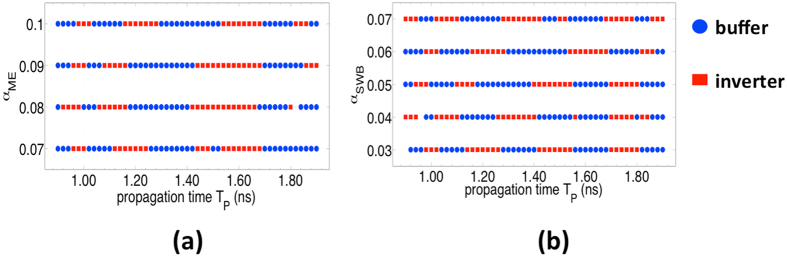
Plot illustrating the sensitivity of the scheme as a function of the damping coefficient *α*_*ME*_ of the ME cell and the propagation time of the spin wave *T*_*P*_. (**a**) Damping coefficient of the PMA SWB *α*_*SWB*_ is kept fixed at 0.06. (**b**) Plot showing the sensitivity of the scheme as a function of *α*_*SWB*_ and *T*_*P*_. *α*_*ME*_ is kept fixed at 0.1. Depending on the choice of *T*_*P*_,ie, the instance at which the voltage is turned off, *α*_*ME*_ and *α*_*SWB*_, we end up with different values of 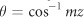 and 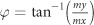 provided by the spin wave as the magnet is switching. Thus for the same input (say, transmission of bit “1”), the same structure acting as a buffer (storage of bit “1”) or an inverter (storage of bit “0”).

In this section, we investigate the effect of choice of *T*_*P*_, *α*_*ME*_ and *α*_*SWB*_ on the device operation. For this we focus only on a single stage comprising of an input ME cell, an output ME cell and an interconnecting SWB. Let us consider the situation when the a spin wave created by the input ME cell has reached the output, but the voltage applied to it is still kept on. The magnetization starts to do a precesion rotation about the out-of-plane (z) axis. As the voltage is turned off, the magnetization follows a simple relaxation dynamics as it falls from out-of-plane to in-plane configuration with initial magnetization angles 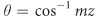 and 
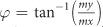
 provided by the spin wave. Depending on the choice of the propagataion time *T*_*P*_,ie, the instance at which the voltage is turned off, *α*_*ME*_ and *α*_*SWB*_, we end up with different values of θ and ϕ for the same input (say, transmission of bit “1”) which results in the same structure acting as a buffer (storage of bit “1”) or an inverter (storage of bit “0”). Figure 5 shows the sensitivity of the scheme to *α*_*ME*_, *α*_*SWB*_ and *T*_*P*_.

### Sequential transmission of information

Next, we investigate our entire three-stage cascaded structure and clocking scheme for sequential transmission of information. By appropriate choice of *T*_*P*_, *α*_*ME*_ and *α*_*SWB*_, we ensure that our structure acts as a chain of inverters. We explore three cases: (a) transmitting all bit “1“s, (b) transmitting all bit “0“s, and (c) transmitting alternately bit “1“s and “0“s. The simulation details and parameters used have been explained under Methods.

**Figure 6:**
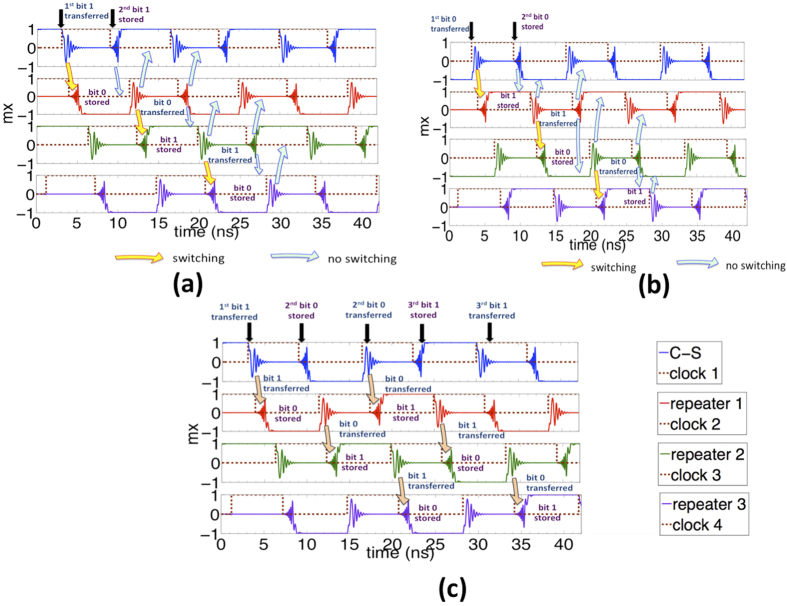
Sequential transmission of information in a clocked three-stage cascaded SWD that acts as a chain of inverters shown in Fig. 1(a). Figure (**a**) (**b**) and (**c**) show the transmission of a train of bit “1”, bit “0” and alternate bits “1” and “0”, respectively. The yellow arrows indicate the case when the spin waves propagate and switch the next stage ME cell while the blue arrows indicate the case when the propagating spin waves do not affect the dynamics of the other ME cells.

Figure 6(a) shows the result of numerical simulation for transmitting a train of bit “1”. We assume the C-S converter is initially storing a bit “1”. As the clock 1 goes high, the magnetization goes out-of-plane creating spin waves and transmitting bit “1” (shown by yellow arrow). The next stage ME cell, ie, repeater 1 is held in the out-of-plane meta-stable state by applying voltage till the spin wave packet arrives. On arrival, clock 2 goes low and it’s magnetization goes in-plane depending on the phase of the wave. We see the transmission of bit “1” successfully switches repeater 1 to mx = −1 final state thus storing bit “0”. As the magnetization of the C-S converter goes in-plane (mx = 1) storing the second bit “1”, this dynamics creates spin waves which do not affect repeater 1 which is in a stable in-plane configuration (shown by blue arrow). Repeater 1 continues to store bit “0” untill clock 2 goes high creating spin waves. These waves propagate in both the directions but do not affect the previous stage ME cell as it is in a stable in-plane configuration (shown by blue arrow). The forward propagating spin waves transmit bit “0” to repeater 2 (shown by yellow arrow). This mechanism goes on thus detecting, storing and transmitting train of bits from one stage to the next. Figures 6(b) show the transmission of a train of bit “0” and alternating bit “1” and “0” respectively in a similar fashion. A similar concept applies to the design of a chain of buffers where a bit “1” gets stored as a bit “1” in the next stage and vice-versa.

## Discussion

The positive or negative current pulses applied to the C-S converter serves as the initial input to the SWD. Thereafter, the information gets encoded either in the phase of the propagating spin waves (phase “0” represents bit “1” while phase “π” represents bit “0”) or as magnetization states (mx = 1 represents bit “1” while mx = −1 represents bit “0”). The scheme of detecting the incoming spin wave signal requires careful selection of the clocking period since depending on the choice of *T*_*P*_ we end up either with a buffer or an inverter as shown in Fig. 5. Note that the reason for a stage acting as a buffer or an inverter lies in the values of magnetization angles *θ* and *ϕ* as the magnet starts to switch. Once we have designed our SWD to act as either of the gates, it is the phase of the spin wave that determines the switching direction, ie, a transmitted bit “1” gets stored as bit “1” (or “0”) while a transmitted bit “0” gets stored as bit “0” (or “1”) in a buffer (or an inverter) structure as is evident from Fig. 6. Our proposal is unique in the sense that in contrast to the earlier proposals of making a buffer or inverter gate by choosing the length of the spin wave interconnect equal to *nλ* or 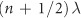 (*λ* being the wavelength of the spin waves and *n* = 1, 2, 3 …)^21^, our scheme provides flexibility in that the same structure can be used as a buffer or an inverter gate by the appropriate choice of the clock period. This serves as a building block for designing majority gates and other logic gates.

In conclusion, we have theoretically demonstrated the possibility of building a complete SWD utilizing the phase of the propagating spin waves for information transission. The proposed charge-to-spin (C-S) converter provides an efficient way for converting information from electrical to spin domain. The spin wave repeaters operating in three different regimes provide a feasible path for spin wave transmission, detection and non-volatile storage. The proposed clocking scheme ensures the sequential transmission of information from one stage to the next while maintaining non-reciprocity. The unique ability to manipulate and use both the amplitude and phase of the spin waves for information transfer and processing in SWD provides an opportunity for building complex logic circuits with fewer logic blocks. However, the most crucial advantage SWD provides over CMOS technology is in terms of power consumption. The integration of the ME cells with the SWB provides a possible route for low power excitation and detection of spin waves and for non-volatile memory element. In this paper, we have provided all the major ingredients for building a complex multi-stage SWD circuitry, including electric-to-spin signal converter (C-S converter), spin wave repeaters that operate in three different regimes of spin wave detection, non-volatile storage and spin wave transmission, spin wave bus interconnect for signal propagation and a novel clocking scheme to enable sequential transmission of information from one stage to the next while ensuring the five essential requirements for logic application: nonlinearity, amplification, concatenability, feedback prevention, and complete set of Boolean operations.

We conclude our work by estimating some of the key characteristics of our proposed SWD and comparing them with the 15 nm CMOS technology and a previously proposed spin wave device (SWD). The data for CMOS (high performance (HP) and low power (LP)) and SWD is based on the benchmarking results presented in 2,3. Table 1 summarizes the data for a FANOUT 1 inverter and a state element (gated D latch). The details of the calculation are shown in section S1 of the supplementary information. It must be noted that the values we provide are an estimate of the performance of the device. We believe further optimization in terms of chosing the proper material parameters can improve the device performance. In spite of being slower than CMOS, SWD holds advantages in terms of area and energy consumption. The improved energy and circuit area and comparable delay for state element makes spin wave logic especially suited for non-volatile computing.

## Methods

### Micromagnetic Simulation

We perform micromagnetic simulations using the Object Oriented Micromagnetic Framework (OOMMF) 27 that numerically solves the modified Landau-Lifshitz-Gilbert (LLG) equation augmented with the spin-transfer-torque term^28^,^29^,^30^. We choose the length of the ME cell to be 160 nm and SWB interconnects to be 500 nm. The width and thickness are fixed at 80 nm and 8  nm respectively. The entire structure is divided into a regular array of meshes of dimension 5 nm × 80 nm × 8 nm. We assume the material of the ME cell FM layer and the SWB to have the same saturation magnetization (*M*_*s*_) of 290 × 10^3^ A/m, exchange stiffness (*A*_*exch*_) of 9 × 10^−12^ J/m. The perpendicular magnetic anisotropy (*K*_*SWB*_) of the SWB is 60 × 10^3^ J/m^3^. The in-plane uniaxial anisotropy for the ME cell FM layer is ignored while the strain-induced perpendicular anisotropy (*K*_*ME*_) is 60 × 10^3^ J/m^3^. A spin-current pulse of *I*_*spin*_ = 0.4 mA and pulse width 0.9 ns is applied locally to the free ferromagnetic layer of the C-S converter. The damping coefficients (α) for the FM layer of ME cell and C-S converter and the SWB is varied as mentioned in the text. To avoid reflections of spin-waves from the boundary, we provide an artificial termination with damping factor α = 1 at the end of the SWB 31. It is to be noted that we have excluded thermal noise in our simulations for simplicity, but the possible effects of thermal fluctuations have been discussed qualitatively in section S4 of supplementary information.

## Author Contributions

S.D., D.E.N, I.A.Y. and A.N. developed the main idea. S.C., N.K. and S.M. contributed to the thermal noise analysis. S.D. performed the simulations,and wrote the paper. All authors discussed the results and agreed to the conclusions of the paper.

## Additional Information

**How to cite this article**: Dutta, S. *et al*. Non-volatile Clocked Spin Wave Interconnect for Beyond-CMOS Nanomagnet Pipelines. *Sci. Rep.* **5**, 9861; doi: 10.1038/srep09861 (2015).

## Figures and Tables

**Table 1:** Estimation and comparison between CMOS, benchmarked SWD and our proposed SWD. The data for CMOS and SWD is based on the benchmarking proposed in [2,3].

		**CMOS HP** 2,3	**CMOS LP** 2,3	**SWD** 2,3	**SWD** (this work)
Inverter with fanout 1	area	0.036 *μ*^*2*^	0.036 *μ*^*2*^	0.0162 *μ*^*2*^	0.0128 *μ*^*2*^
	energy	31.2 aJ	5.27 aJ	4.51 aJ	26 aJ
	delay	0.78 ps	97.6 ps	0.45 ns	2 ns
State element	area	0.648 *μ*^*2*^	0.648 *μ*^*2*^	0.0162 *μ*^*2*^	0.0128 *μ*^*2*^
	energy	0.45 fJ	76.8 aJ	4.51 aJ	26 aJ
	delay	9.73 ps	1.2 ns	0.45 ns	2 ns
first stage optional input*	energy	—	—	—	7 fJ

^*^STT used in C-S converter for storing either bit 1 or 0.
